# Implementation of the equivalent temperature measurement system as a part of the vehicle Heating, ventilation and Air-conditioning unit

**DOI:** 10.1186/2046-7648-4-S1-A159

**Published:** 2015-09-14

**Authors:** Jan Fišer, Aleš Povalač, Tomáš Urbanec, Jan Pokorný, Miloš Fojtlín

**Affiliations:** 1 Department of Thermodynamics and Environmental Engineering, Energy Institute, Faculty of mechanical engineering, Brno University of Technology, Czech Republic

## Introduction

Thermal comfort evaluation based on the Comfort zone diagram is relatively new and promising method [1] developed by Håkan O. Nilsson [2]. The method was developed mainly for non uniform indoor environments [3] such as vehicle cabins [4]. Mean thermal vote (MTV) is correlated with equivalent temperature, which is typically measured by a thermal manikin with clothing or by a sensor with heated surface. This fact is the advantage of this method because prediction of thermal comfort is based on a measurable physical phenomenon which is called dry heat loss. The essence of this method inspired us to develop a measurement system that will be based on miniaturised and cost effective equivalent temperature sensors. Such sensors could be easily integrated into the surroundings of seated human and could provide data about local thermal comfort as feedback information for HVAC control unit. Our project, which started last year, is called Innovative control for Heating, Ventilation and Air Conditioning systems, iHVAC.

## Methods

**Figure 1:**
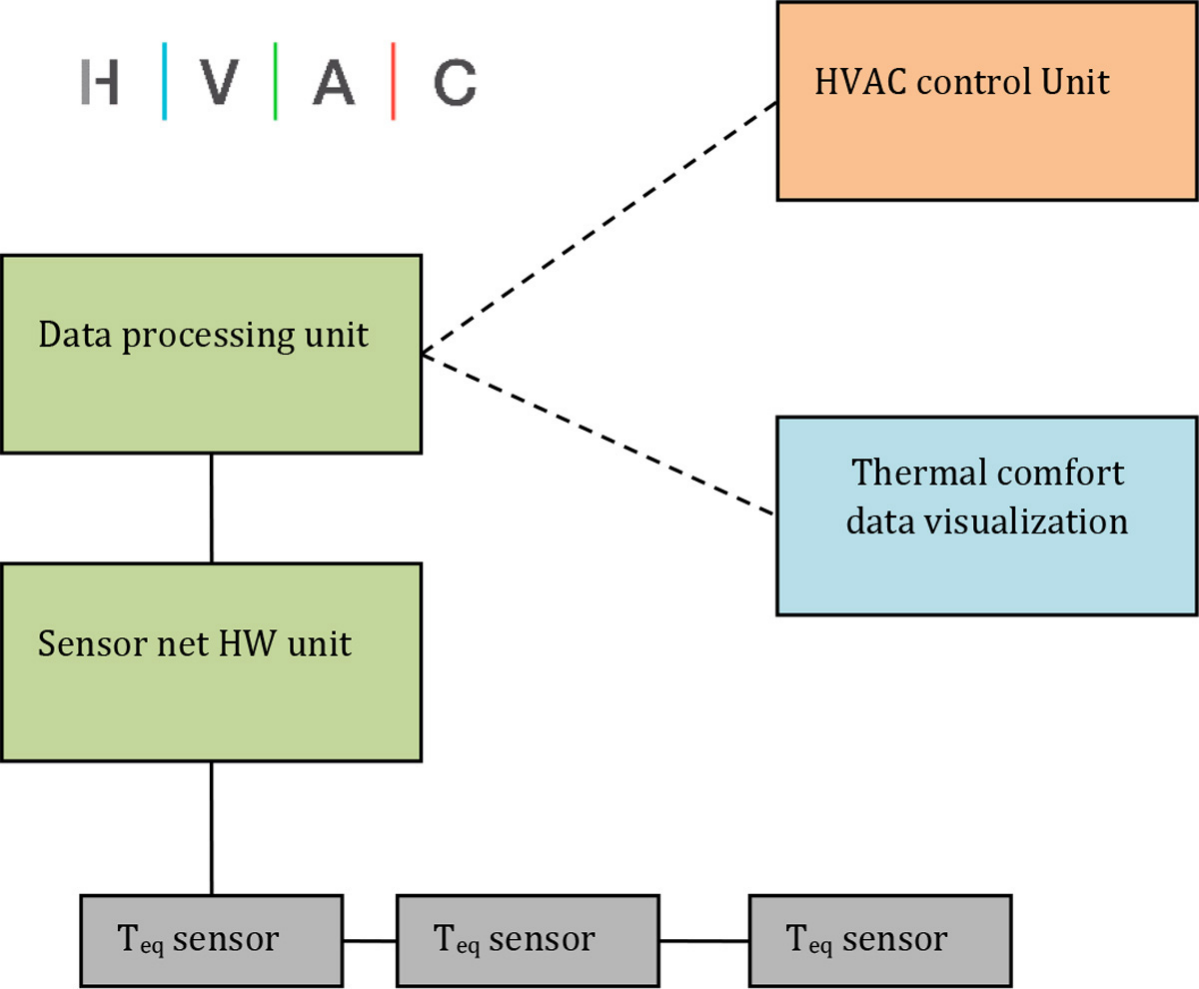
**The main parts of the measurement system based on equivalent temperature sensors**.

The functional scheme of the proposed sensing system that measures equivalent temperature and links the data from the sensors to thermal comfort is shown in Figure 1. It contains the following parts: the net of T_eq _sensors, the main hardware units for sensor net controlling and data processing, the data interfaces connected to the HVAC control unit and the data visualization unit. The sensors have to be calibrated first to obtain a calibration function that describes the relation between a surface temperature of the sensor and the corresponding equivalent temperature. Another reason for the calibration is the fact that the typical control mode of the sensor is constant heat flux thus the surface temperature of the sensor is a result of surrounding environmental parameters and the Teq cannot be calculated without calibration function/curve.

## Conclusion

The iHVAC project is now in a phase of hardware and software demonstrator system development. In following two years a phase of integration, calibration and validation with test subjects is planned. The main aim of the project is to demonstrate that the relatively simple measurement system can supply relevant data about local thermal comfort. If the validation is successful then the system will provide useful feedback for the HVAC control unit. We expect more accurate control inputs of the HVAC system regarding the individual passenger needs, as well as resulting in lower energy demands.

## Acknowledgements

The research is supported by the project Innovative Control of HVAC as the Part of Driver Assistance System, TA04031094.
